# Indications, procedural standards, and training requirements for flexible nasolaryngoscopy in pulmonology – a Delphi study protocol^✰^

**DOI:** 10.1016/j.mex.2026.104191

**Published:** 2026-10-07

**Authors:** Ajit Nair, Philip G Bardin, Paul Leong

**Affiliations:** a Monash Lung Sleep Allergy and Immunology, Monash Health, Clayton, Victoria, Australia; b School of Clinical Sciences, Monash University, Clayton, Victoria, Australia; c Department of Internal Medicine, Latrobe Regional Health, Traralgon, Victoria, Australia; d Hudson Institute, Monash University, Melbourne, Australia

**Keywords:** Laryngoscopy, Fiberoptic, Respiratory tract diseases/diagnosis, Pulmonary medicine/education, Clinical competence

## Abstract

Flexible nasolaryngoscopy (FNL) is a well-established, minimally invasive procedure for assessing the nasal passages, nasopharynx, and larynx. Its role in pulmonology is expanding, particularly for evaluating vocal cord dysfunction/inducible laryngeal obstruction, chronic cough, and other upper airway conditions relevant to the “united airways” model of care. However, no consensus-based guidance currently exists on the indications, procedural standards, or training requirements for FNL performed by pulmonologists. This protocol describes a modified two-round Delphi study that will survey an international, purposively sampled panel of pulmonologists and otolaryngologists via an online platform hosted by Monash University, using a five-point Likert scale and pre-specified consensus thresholds (≥70% agreement). Findings will be discussed in a structured expert roundtable to support interpretation and contextualization. • Establishes expert consensus on clinical indications and contraindications for FNL in pulmonology practice. • Develops consensus on procedural standards, curriculum, and competency requirements for FNL training in pulmonology. • Identifies priority areas for future research to support integration of FNL into pulmonology services.

## Graphical abstract

**Figure:**
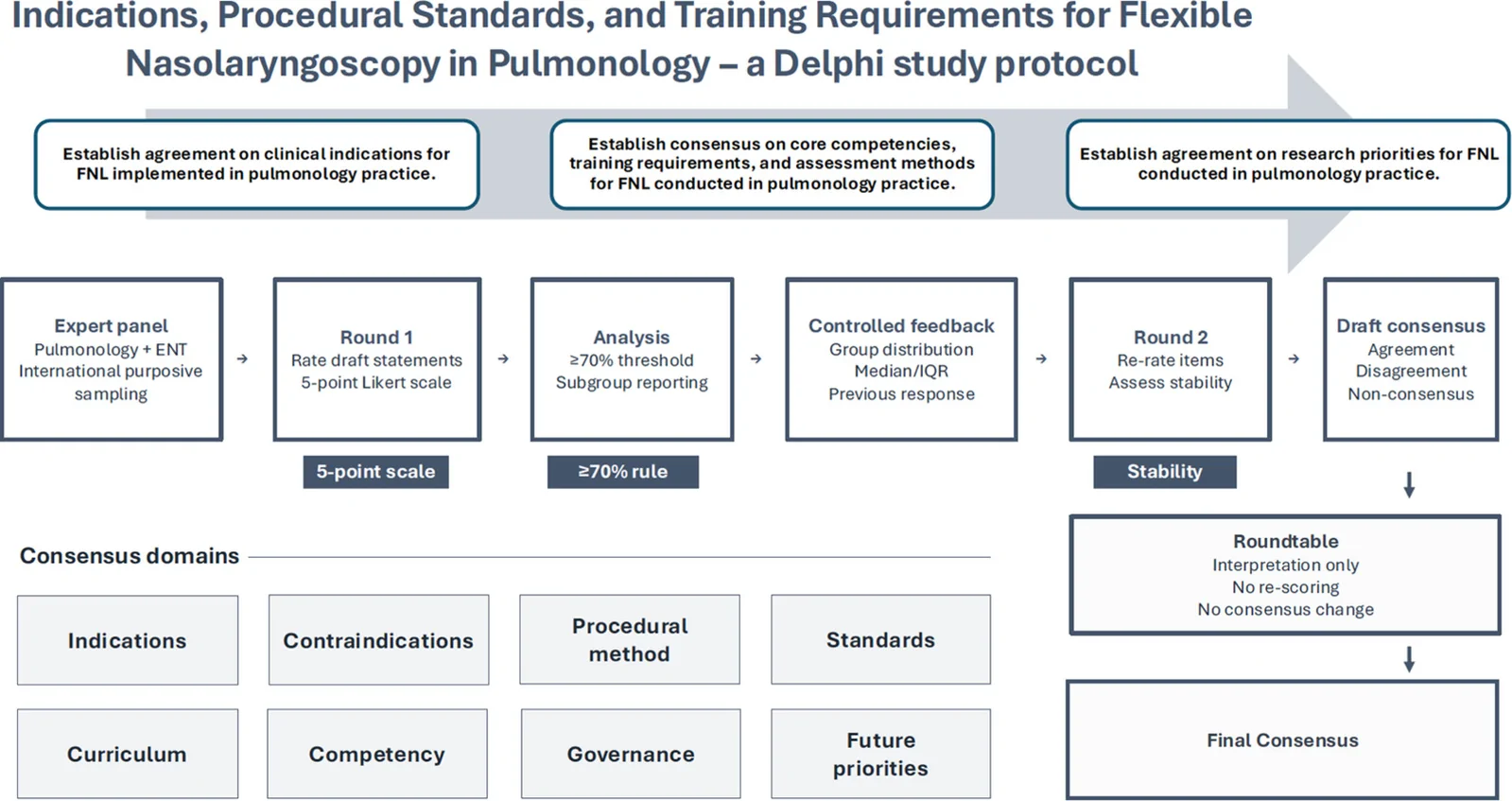

## Specifications table

**Table:** 

**Subject area**	Medicine and Dentistry
**More specific subject area**	Laryngoscopy, Fiberoptic; Respiratory Tract Diseases/Diagnosis; Pulmonary Medicine/Education; Clinical Competence
**Name of your protocol**	Indications, Procedural Standards, and Training Requirements for Flexible Nasolaryngoscopy in Pulmonology – a Delphi study protocol
**Reagents/tools**	REDCap, hosted by Monash University
**Experimental design**	Modified two-round Delphi methodology, followed by a structured online expert roundtable.
**Trial registration**	Submitted for registration with ANZCTR
**Ethics**	Monash Health HREC A: RES-26-0000-089A Records will be maintained in a secure password protected digital format conforming to the 2019 National Health and Medical Research Council Management of Data and Information in Research: A guide supporting the Australian Code for the Responsible Conduct of Research. These will be held for 7 years and will be accessible only to authorised personnel. Responses will be held on a secure two-factor authenticated Monash University platform.
**Value of the Protocol**	• Provides the first expert consensus-based indications and procedural standards for FNL performed in pulmonology practice. • Establishes a structured foundation for competency-based training, assessment, and curriculum development for pulmonologists performing FNL. • Supports safe, standardised, and appropriately governed implementation of FNL services, aligned with the “united airways” model of integrated airway care.

## Background

Flexible nasolaryngoscopy (FNL) is a minimally invasive, low‑risk procedure used to visualise the nasal passages, nasopharynx, and larynx. It is an essential procedure with findings that can critically influence diagnosis, prognosis, and management of many pathologies and diseases. Over the past several decades, advances in fibreoptic technology have transformed upper airway assessment from rigid to flexible endoscopy [1,2], greatly improving patient tolerability and enabling routine outpatient use in a range of clinical settings. Growing acceptance of the “united airways” concept, which views the upper, middle and lower respiratory tract as a continuous, interrelated system, has driven integrated, cross‑specialty approaches to airways disease [3] and required extension of the scope of FNL.

Although FNL has predominantly been the purview of otolaryngology, its role in pulmonology is expanding, principally in the evaluation of vocal cord dysfunction/inducible laryngeal obstruction (VCD/ILO) but also in chronic rhinosinusitis with nasal polyposis (CRSwNP), obstructive sleep apnoea (OSA), haemoptysis or epistaxis, laryngopharyngeal reflux (LPR), and chronic cough. Historically, respiratory training has placed limited emphasis on systematic upper/middle airway examination, yet as pulmonologists increasingly adopt treatable traits [4] and implement precision-based models of care, incorporating direct upper and middle airway inspection into routine assessment is becoming increasingly important.

A prior Delphi study in otolaryngology (ENT) has defined key indications and technical standards for FNL and informed the development of a structured procedural assessment tool [5]. However, comparable guidance tailored to the practice of pulmonology is lacking and the aim of this Delphi study is therefore to develop expert consensus on the indications, procedural standards, and training requirements for FNL in pulmonology. The goal is to support safe, effective, and appropriately governed implementation of this procedure and to provide a foundation for competency-based training, assessment, and service design in pulmonology.

## Description of protocol

### Study objective

The primary aims of this study are to:

1. Establish agreement on clinical indications for FNL implemented in pulmonology practice.
2. Establish consensus on core competencies, training requirements, and assessment methods for FNL conducted in pulmonology practice.
3. Establish agreement on research priorities for FNL conducted in pulmonology practice.

### Methods

This study will use a modified two-round Delphi methodology [6] to develop expert consensus on the indications, procedural standards, and training requirements for FNL in respiratory and sleep medicine. The Delphi technique is a structured method that gathers and refines expert opinion through multiple survey rounds with controlled feedback, while maintaining anonymity between participants to reduce the influence of dominant individuals and group pressure [7]. A modified Delphi approach is particularly appropriate when there is an emerging evidence base and substantial experiential knowledge, but no established guideline or standard, as is the case for FNL in pulmonology [8].

### Study schema

The Delphi will be performed via REDCap, hosted by Monash University. Prospective participants will receive an email invitation describing the study objectives, procedures, and including a link to the survey. The email will also contain a link to the participant information and consent form. Commencement of the survey will be taken as an indication of participant provision of implied consent. Participants completing Round 1 will be eligible for Round 2. Round completion will be defined prospectively, and item-level consensus calculations will use the number of participants providing a substantive rating for that item; “unable to judge/not applicable” responses will be reported separately.

A structured literature-informed and purposive sampling strategy will be used to identify potential expert panelists for the modified Delphi process. A targeted literature search will be conducted to identify authors with expertise in FNL, airway endoscopy, and procedural training, and first, senior, and corresponding authors will be extracted as potential candidates. Searches will be limited to human studies published in English within the preceding 10 years. Additional experts will be identified through authorship of relevant clinical guidelines and consensus statements, leadership roles within professional societies, and recommendations via snowball sampling from initial invitees. Reliance in part on English-language publications from the preceding 10 years may under-identify experts from non-English-speaking regions; to reduce this bias, the literature search will be only one of several recruitment pathways, alongside guideline authorship, professional society involvement, direct regional outreach, and snowball sampling, and society contacts will be specifically asked to nominate colleagues whose relevant expertise may not be well represented in the English-language literature.

Eligible panelists will be required to meet all the following core criteria:

1. Specialist qualification in respiratory medicine/pulmonology or otolaryngology (ENT)
2. A minimum of five years of independent post-fellowship clinical experience
3. Active clinical involvement in airway disease and/or endoscopic procedures relevant to FNL

In addition, individuals will be required to meet at least two of the following expertise criteria:

1. Regular performance of airway endoscopic procedures (e.g., bronchoscopy, laryngoscopy, or related techniques)
2. Formal involvement in clinical education or training
3. Peer-reviewed publications in airway disease, endoscopy, or medical education
4. Participation in guideline development or professional societies
5. Experience in competency-based training or assessment.

Two complementary forms of expertise are represented within the panel. Technical FNL experts have current experience performing or directly supervising FNL and will contribute particularly to statements concerning procedural technique, anatomy, safety, and competency. Pulmonology implementation/training experts may not routinely perform FNL but must have substantive clinical, educational, or service-development expertise relevant to airway disease, procedural training, or implementation of FNL within pulmonology practice.

Procedure-specific experience, including whether participants currently perform or supervise FNL and their approximate procedural volume, will be collected using categorical responses. Participants without relevant experience for a particular technical item will be able to select “unable to judge”, and these responses will not contribute to the denominator used to determine consensus for that item.

Eligibility will be assessed before invitation using information from professional profiles, publications, and guideline or professional society roles, together with a brief eligibility assessment confirming clinical experience and relevant expertise. Eligibility will be independently reviewed by two members of the study team, with any disagreement resolved by discussion with a third investigator. Procedural volume will not be used to weight individual participants’ responses, as there is no validated procedural-volume threshold that would justify differential weighting of expert opinion.

A stratified purposive sampling approach will be used to ensure balanced representation across specialties (ENT and pulmonology), pulmonology subspecialties (including airways disease and interventional pulmonology), and geographic regions (Australia/New Zealand, North America, Europe, Asia, Africa, the Middle East, and South/Central America). ENT specialists are intentionally included to provide complementary expertise in upper airway anatomy, pathology, and endoscopic technique, thereby strengthening the validity of procedural and safety recommendations. A fixed recruitment quota will target approximately 60–65% pulmonology and 35–40% ENT participation among panel completers, consistent with the intended focus of recommendations on pulmonology practice. Approximately 30–40 experts will be invited with the aim of achieving a final panel of 20–30 participants completing all Delphi rounds, allowing for expected attrition during the Delphi process. All invited participants will be assessed against the predefined criteria prior to inclusion.

• Recruitment targets will be set by specialty and region, in keeping with the sampling approach above, with a minimum of 2–3 completers sought from each regional stratum.
• A minimum final panel of 15 will be required. If this is not achieved, recruitment will be extended rather than proceeding with an inadequately sized panel.
• Each Delphi round will remain open for approximately three weeks, with reminder emails sent to non-responders at days 7 and 14.
• Participants who do not complete Round 1 within the response window will not be invited to Round 2. Attrition between rounds will be reported by specialty and region so that any resulting imbalance is clear.

### Participant identification, confidentiality, and acknowledgement

Each participant will be assigned a unique study ID by the research team. The study ID will be used to link Round 1 and Round 2 responses, monitor completion of both rounds, and manage withdrawal requests where this remains possible. The study ID key linking participant names/contact details to survey responses will be stored separately from the survey dataset in a secure, password-protected Monash University platform accessible only to authorised study personnel.

Survey responses will be analysed and reported in aggregate form. Individual responses and free-text comments will not be attributed to named participants in publications or presentations. However, participants who complete both Delphi rounds may be invited to be acknowledged or included in a group authorship by-line in resulting publications, with their agreement. For this reason, participation in the study is not fully anonymous to the research team, and acknowledged participants may be identifiable as members of the expert panel in publications. This will be made clear in the Participant Information and Consent Form and invitation materials. Participants may opt out of acknowledgement or group authorship without penalty.

### Questionnaire development

Round 1 items were derived from a companion narrative review, “Flexible Nasolaryngoscopy for Pulmonologists” (Nair et al., under review). The review comprised a structured MEDLINE search (via Ovid/PubMed) from database inception to 30 November 2025, using seven concept-specific search blocks covering vocal cord dysfunction/inducible laryngeal obstruction (VCD/ILO), laryngopharyngeal reflux, chronic rhinosinusitis with nasal polyposis, obstructive sleep apnoea, chronic cough, and training/competency literature, each combined with an endoscopy/laryngoscopy-specific term to limit retrieval of unrelated intubation/difficult-airway literature, and limited to English-language human studies (2578 records identified). This was supplemented by targeted searching of specialty society and college websites, guideline repositories, and reference lists of key articles. Title/abstract screening was performed by one author (AN), with full-text review where relevance was unclear from the abstract, consistent with a narrative rather than systematic review.

Extracted evidence from the narrative review, together with the prior ENT-based Delphi instrument, was mapped to candidate Round 1 statements in an evidence-to-statement table reviewed by the full co-investigator team before finalisation. The item-development evidence base includes direct ENT input, as the narrative review’s author group includes an ENT co-author.

In this protocol, “middle airway” denotes the vocal folds and adjacent laryngeal structures as a functional zone distinct from the upper airway (nose to oropharynx) and the lower/tracheobronchial airway, consistent with established usage in the pulmonology and laryngology literature [9,10].

### Delphi rounds

Round 1 will examine views on respiratory FNL. A focused narrative review of FNL indications and training for pulmonologists has been undertaken and a Delphi on development of a procedure specific assessment tool for FNL has been reported [5] and validated in ENT [11]. Statements will be presented to participants for rating using a five-point Likert scale (1 strongly disagree to 5 strongly agree) [Appendix 1]. Participants will also be invited to contribute free text items which may be presented to the group in Round 2.

In Round 2, participants will re-rate all statements after reviewing their own previous rating and group distribution.​ Stability of responses will be assessed; items achieving consensus are classified as final recommendations; persistent non-consensus is documented. New statements from qualitative content analysis of Round 1 will be presented (anonymised).

Following completion of the final Delphi round, the draft set of indications, procedural standards, and training requirements for flexible nasolaryngoscopy will undergo structured interpretation and contextualization via an online expert roundtable.

For each repeated item in Round 2, participants will receive feedback comprising:

• Their own previous response
• The overall distribution of Round 1 responses
• The median and interquartile range
• Specialty-specific response distributions
• A concise anonymised summary of relevant free-text comments

Free-text responses will be reviewed independently by two investigators. Suggestions will be grouped within the existing Delphi domains, with similar or overlapping suggestions combined where appropriate. New statements will be added if they introduce a distinct concept, are relevant to the scope of the study, and can be clearly rated as a standalone item. Any differences in interpretation, inclusion, or wording will be resolved by discussion, with a third investigator involved if needed.

All Round 1 statements will be re-rated in Round 2, including those that already reach consensus, to assess the stability of responses between rounds. Wherever possible, the wording will remain unchanged. If feedback from Round 1 identifies a genuine ambiguity that requires substantial rewording, the revised statement will be treated as a modified or new item and excluded from the formal between-round stability analysis for the original statement.

The purpose of the roundtable will be to:

• Review areas of persistent non-consensus;
• Discuss interpretation and practical implementation of the final recommendations; and
• Identify priorities for future research and curriculum development.

The roundtable will be conducted online via a secure videoconferencing platform (e.g. Zoom) and is anticipated to last approximately 60 min. Participation will be voluntary and limited to Delphi participants who indicate willingness to attend. Attendance will not be mandatory for inclusion in the Delphi panel or group authorship of resulting publications.

No additional quantitative data collection or re-scoring of Delphi items will occur during the roundtable. Feedback obtained during the session will be used descriptively to assist interpretation and contextualisation of the final consensus findings. Any comments included in publications will be de-identified and reported in aggregate form.

There will be no patient involvement as this is solely healthcare practitioner research.

### Analysis

Statistical analysis will be performed using standard statistical software (e.g. SPSS). At the end of the final round, all items will be grouped into four categories (consensus agreement, consensus disagreement, consensus neutral, and no consensus) and presented by domain (indications, contraindications, procedural standards, procedural methods, curriculum, competency, governance, future priorities).

For each round and each item, the following will be calculated: frequency and percentage for each response option on the 5-point Likert scale, median, and interquartile range. Consensus status (agreement, disagreement, neutral, or no consensus) will be determined according to the pre-specified thresholds. Response rates per round and patterns of item completion will also be summarised. Where panel size permits, exploratory subgroup analyses will be conducted using appropriate non-parametric tests to identify meaningful differences in ratings, acknowledging the study is not powered for formal hypothesis testing. Free‑text responses from each round will undergo qualitative content analysis.

The IQR will be used descriptively to summarise dispersion of panel ratings for each item and as one component of the assessment of response stability between rounds. Consensus will be determined primarily by the pre-specified percentage thresholds below, rather than by IQR alone. However, IQR will be reported to assist interpretation of the strength and spread of expert agreement.

Response stability between Round 1 and Round 2 will be assessed for each repeated item using:

• Change in median Likert score between rounds
• Change in the percentage of panelists rating the item in the consensus direction (agreement, disagreement, or neutrality); and
• Change in IQR between rounds.

An item will be considered stable if the median rating is unchanged between rounds, the percentage agreement/disagreement/neutrality in the relevant direction changes by 10 percentage points or less, and the IQR does not widen by more than 1 point. Items that newly achieve consensus in Round 2 will be classified according to the consensus thresholds above. Items that remain below consensus thresholds despite stable responses will be reported as stable non-consensus items. Items with unstable responses will be described as unresolved and interpreted cautiously.

Reporting will follow recommended guidance for transparency in consensus and Delphi methodology in health services research [12], including clear description of panel composition, response rates, consensus thresholds, and handling of non‑consensus items.

### Definition of consensus

Consensus in this Delphi study will be defined using explicit quantitative thresholds consistent with established approaches in health‑care consensus methods [8,13].

Agreement will be defined as ≥ 70% of the expert panel rating preferences as follows:

• When ≥70% of the expert panel indicates Likert scores of 4 or 5 (corresponding to “agree” or “strongly agree”, “important” or “very important” or similar) for a given statement, a positive consensus has been attained. For dichotomous questions, ≥70% of the expert panel indicating “yes” will be regarded as a positive consensus response.
• When ≥70% of the expert panel indicates either Likert scores of 1 or 2 (corresponding to “disagree” or “strongly disagree”, “not all important” or “low importance”, or similar) for a given statement, a negative consensus has been attained. For dichotomous questions, ≥70% of the expert panel indicating “no” will be regarded as a negative consensus response.
• When ≥70% of the expert panel indicates Likert 3 (“neutral”) for a given statement, a consensus for neutrality has been attained.
• When none of the three preceding definitions for consensus are met, the statement is classified as having no consensus.

Consensus will additionally be assessed and reported at two levels for every item: the pooled panel using the threshold above, and separately within the pulmonology and ENT subgroups. An item will be classified as a final consensus recommendation only if the pooled threshold is met and the pulmonology subgroup independently meets the same threshold in the same direction. Items that reach pooled consensus without independent pulmonology-subgroup consensus will be classified as “specialty-discordant”, reported with specialty-stratified frequencies, medians, and interquartile ranges shown side by side, and referred to the expert roundtable for discussion rather than reported as an agreed recommendation. Item-level consensus calculations will exclude “unable to judge/not applicable” responses from the denominator.

### Protocol contextualisation

As this is a study protocol, outcome data are not yet available. Content and face validity of the Round 1 survey items were informed by a focused narrative review of the FNL literature and by the prior ENT-based Delphi study of a procedure-specific FNL assessment tool [5], and the item set will be reviewed by the co-investigator team prior to distribution. Following completion of both Delphi rounds, an online expert roundtable will be used to support the interpretation and contextualisation of the consensus findings, including areas of persistent non-consensus, practical implementation considerations, and priorities for future research.

### Limitations

• As a consensus-based methodology, Delphi findings reflect expert opinion rather than experimentally validated clinical outcomes.
• Despite a structured, literature-informed and purposive sampling strategy, selection of panelists may introduce sampling bias.
• Although geographic representation across Australia/New Zealand, North America, Europe, Asia, Africa, the Middle East, and South/Central America will be sought, reliance in part on English-language, literature-based identification of experts may still under-represent some regions, and findings may not generalise to all healthcare settings, including lower-resource contexts.
• Attrition between Rounds 1 and 2 could affect the representativeness of the final consensus recommendations.
• There is no direct patient or consumer involvement in the consensus process as this is healthcare practitioner research

## CRediT authorship contribution statement

**Ajit Nair:** Conceptualization, Methodology, Investigation, Writing – original draft. **Philip G Bardin:** Conceptualization, Supervision, Writing – review & editing. **Paul Leong:** Conceptualization, Methodology, Supervision, Writing – review & editing.

## Declaration of competing interest

The authors declare the following financial interests/personal relationships which may be considered as potential competing interests:

AN has no disclosures of interest.

PL and PGB are non-compensated Board members of Global Initiative for Inducible Laryngeal Obstruction (GIILO).

## Data Availability

No data was used for the research described in the article.

## Appendix Supplementary materials

Supplementary material *and/or* additional information Appendix 1: Round 1 Delphi survey (demographics and Sections A–H)
