# Supplementary material for: Indications, procedural standards, and training requirements for flexible nasolaryngoscopy in pulmonology – a Delphi study protocol
Source: MethodsX. 2026 Oct 7;17:104191. doi: 10.1016/j.mex.2026.104191 (PMC13677959; doi:10.1016/j.mex.2026.104191)
Supplement: Supplementary file 1 [file mmc1.docx]

**Appendix 1: Round 1 Questions**

Unless otherwise specified, items are rated on a 5-point agreement scale (1 = strongly disagree, 2 = disagree, 3 = neutral, 4 = agree, 5 = strongly agree). Where importance rather than agreement is being judged (Section H), the same 5-point scale is used with anchors 1 = not at all important to 5 = very important. Where essentiality is being judged (Section F), the scale anchors are 1 = not essential to 5 = essential. An “unable to judge / not applicable” option is available for every item and is excluded from the denominator used to determine consensus for that item.

In this survey, “upper airway” refers to the nasal cavity, nasopharynx, and oropharynx, and “middle airway” refers to the vocal folds and adjacent laryngeal structures, consistent with established usage in the pulmonology and laryngology literature. “Routine awake FNL” refers to standard diagnostic FNL performed at rest; “provocation laryngoscopy” and “continuous laryngoscopy during exercise (CLE)” are distinct techniques used specifically to investigate exercise-related breathlessness and are identified separately where relevant below.

**Demographics**

**What is your age band?**

Checklist: <35, 35–44, 45–54, 55–64, ≥65

Please indicate your diploma and qualifications.

Checklist: MBBS/MD, PhD, B.App.Sc, Other (specify)

**Please indicate your practice environment (tick all that apply).**

Checklist: community, academic hospital, research, private practice, regional hospital, low-resource/limited-specialist-access setting, other (specify)

**Please indicate your country/region of practice.**

Checklist: Australia/New Zealand, North America, Europe, Asia, Africa, Middle East, South/Central America, Other (specify)

**Please indicate your practice specialty.**

Checklist: Respiratory/pulmonology, sleep medicine, allergy/immunology, otolaryngology (ENT), other (specify).

**How many years ago did you complete your clinical training?**

**Approximate number of FNL procedures you personally perform or directly supervise per month.**

Checklist: 0 (do not currently perform/supervise), 1–4, 5–10, >10 per month

**Do you supervise trainees performing FNL?**

Yes/No

**Have you participated in, or been an investigator for studies of FNL?**

Yes/No

**Approximately how many scientific publications have you authored:**

- FNL? (categories: 0, 1–5, 6–15, >15)
- In total (including FNL)? (categories: 0, 1–5, 6–15, >15)

**Have you ever received funding for FNL research?**

Yes/No

**Do you have a financial conflict of interest regarding FNL?**

Yes/No

**Indications, Procedural Standards, and Training Requirements for Flexible Nasolaryngoscopy in Pulmonology Delphi**

**Section A: Indications and clinical use**

1. FNL should be available within pulmonology services as a routine diagnostic tool for upper airway assessment.
2. FNL is an appropriate tool for evaluating unexplained stridor or suspected upper airway obstruction in pulmonology clinics.
3. FNL is an appropriate tool for evaluating chronic cough when upper/middle airway pathology is suspected.
4. FNL is an appropriate tool for evaluating suspected vocal cord dysfunction/inducible laryngeal obstruction (VCD/ILO) in pulmonology.
5. FNL (including provocation laryngoscopy or continuous laryngoscopy during exercise, where clinically indicated) is an appropriate tool for evaluating exercise-related breathlessness not yet attributed to a specific diagnosis.
6. FNL is an appropriate tool for evaluating upper/middle airway manifestations (e.g. nasal polyps) of type 2 inflammation in airways disease.
7. FNL findings may contribute, as part of a multimodal assessment, to the evaluation of suspected laryngopharyngeal reflux (LPR) in patients seen in pulmonology clinics; laryngoscopic findings alone should not be used to establish an LPR diagnosis.
8. FNL should be considered for pre‑procedural assessment in patients undergoing planned upper airway or thoracic interventions (e.g. bronchoscopy, tracheostomy assessment) when laryngeal pathology is suspected.
9. FNL is appropriate for follow‑up of known non-malignant laryngeal pathology in collaboration with ENT colleagues when agreed care pathways exist.
10. FNL should not be performed in pulmonology clinics without ready access to appropriate escalation pathways for abnormal findings.
11. Please list any additional indications or situations where you believe FNL should or should not be used in pulmonology practice.

**Section B: Contraindications**

1. FNL should generally be avoided in patients with severe, acute respiratory distress where airway compromise is imminent, unless performed in a setting with full airway support.
2. FNL should generally be avoided in patients with known or suspected unstable cervical spine injury unless cleared by appropriate specialists.
3. FNL should generally be avoided in patients with inability to cooperate (e.g. severe agitation) unless adequate support, monitoring, and expertise are available.
4. FNL can be safely performed in patients on therapeutic anticoagulation with appropriate risk assessment and local protocols.
5. A standardised checklist for FNL patient selection (including comorbidities, anticoagulation, and airway risk) should be used in pulmonology clinics.
6. Please suggest any other key contraindications or selection criteria you consider important.

**Section C: Procedural standards**

1. FNL in pulmonology clinics should be performed in a dedicated procedure room or clinic area with access to resuscitation equipment.
2. Staff performing or supervising FNL should have documented training in basic airway management and recognition of complications.
3. If used, topical anaesthesia and nasal decongestion should follow a standard protocol (drug choice, dose limits and contraindications) within the service.
4. Local infection‑prevention policies for endoscope reprocessing and high‑level disinfection should be explicitly applied to FNL performed in pulmonology services.
5. A structured pre‑procedure safety pause (time‑out) should be used before FNL (confirm patient identity and indication).
6. Services performing FNL should maintain a log of procedures and complications (e.g. epistaxis, laryngospasm, vasovagal events).
7. Written post‑procedure information (including red‑flag symptoms and contact details) should be routinely provided to patients.
8. Please list additional safety or environmental standards you consider essential for FNL in pulmonology services.

**Section D: Procedural method**

1. A standardised stepwise approach to FNL (nasal passage, nasopharynx, oropharynx, larynx, and subglottis where adequately visualised) should be described in local protocols.
2. Video recording and/or still image capture should be available for documentation and teaching where feasible, with appropriate consent and data governance.
3. A structured report template should be used for FNL, including anatomical description, dynamic findings, and clinical interpretation.
4. Use of standardised grading or scoring systems (e.g. for vocal cord movement and laryngeal inflammation) should be encouraged to improve communication between specialties.
5. Documentation should clearly indicate the level of supervision (e.g. trainee under direct supervision, independent specialist).
6. Please suggest any additional standards for FNL technique or reporting that you consider important.

**Section E: Curriculum**

1. Pulmonologists who perform FNL independently should complete a formal training program, where feasible, that includes didactic teaching, simulation (where available), and supervised procedures.
2. Trainees should perform a minimum number of supervised FNL procedures before being considered for independent practice (please rate the importance of having a minimum number, not the specific number).
3. A logbook of FNL procedures (including indication, findings, complications, and supervision level) should be mandatory for trainees.
4. Simulation‑based training (e.g. task trainers, virtual reality, or high‑fidelity models) should be incorporated into FNL training where available.
5. Initial training in FNL for pulmonologists should involve collaboration with ENT specialists where feasible.
6. Periodic refresher training (e.g. workshops, video‑based review of complications and errors) should be recommended for clinicians performing FNL.
7. If you believe a minimum case number is essential, what range would you recommend before independent practice (e.g. 10–20, 21–40, >40)?
8. Please describe any additional training pathway elements you consider important.

**Section F: Competency**

For the following, rate how essential each competency or assessment method is for sign‑off to independent practice.

1. Knowledge of relevant upper/middle airway anatomy and common laryngeal pathology.
2. Ability to counsel patients about indications, risks, benefits, and alternatives to FNL.
3. Technical ability to pass the scope atraumatically through the nasal passage and complete a systematic examination of the larynx.
4. Ability to recognise and respond appropriately to patient discomfort or distress during FNL.
5. Ability to recognise and manage common complications (e.g. minor epistaxis, vasovagal reactions) and to escalate care when needed.
6. Ability to interpret dynamic findings relevant to pulmonology (e.g. paradoxical inspiratory vocal cord movement, concentric supraglottic collapse).
7. Use of a validated structured procedure‑specific assessment tool (see below) for formative assessment during training [5][9].
8. Use of at least one summative direct observation before sign‑off for independent practice.
9. Multisource feedback as part of FNL competence assessment is desirable.
10. Please list any additional competencies or assessment strategies that should be included in an FNL training framework for respiratory and sleep medicine.

**FNL Structured Formative Assessment Tool**

| **Item** | **Poor performance** | | **Average performance** | | **Good performance** | |
| --- | --- | --- | --- | --- | --- | --- |
| Optimizes the positioning of the patient when needed to achieve the best possible conditions for the examination. | Omits positioning of the patient completely. |  | Positions the patient in an insufficient manner or fails to maintain positioning throughout the examination. |  | Secures and maintains an optimal positioning throughout the examination |  |
| Utilizes a gentle smooth movement of the endoscope. | Clumsy motion, many unnecessary movements. |  | Stable motion, some unnecessary movements. |  | Fluent motion. No unnecessary movements. |  |
| Maintains a clear field of vision when possible. | Many redouts/whiteouts. |  | Some redouts/whiteouts. |  | No redouts/whiteouts. |  |
| *(Optional)*  *Performs maneuvers intended to clear mucus/fog from endoscope tip.* | *Fails to react to substantial obstruction of visualization due to mucus/fog.* |  | *Makes relevant but insufficient attempts at clearing mucus/fog.* |  | *Makes relevant and successful attempts at clearing mucus/fog from endoscope (including if needed extracting the endoscope to clean it).* |  |
| Applies anatomical knowledge when navigating narrow or sensitive areas (i.e. septum vs turbinate, posterior wall vs epiglottis, vocal folds). | Performance reveals a lack of understanding i.e. undue pressure against incompliant structures or failure to avoid sensitive areas. |  | Performance reveals some understanding but with some pressure against incompliant or sensitive structures. |  | Performance reveals a good understanding i.e. no undue pressure against incompliant structures and deliberate avoidance of contact with sensitive areas (i.e. septum vs turbinate, epiglottis vs posterior wall, vocal folds). |  |
| To the extent possible, visualizes all mucosal surfaces of the nasopharynx (postero-superior wall, lateral wall including the fossa of Rosenmüller, inferior wall (soft palate). | Fails to visualize relevant structures. |  | Visualizes most relevant structures but in a hurried or “once over lightly” fashion. |  | Visualization of all relevant structures executed without negligence or omissions. |  |
| To the extent possible, visualizes all mucosal surfaces of the oropharynx (anterior wall including the base of tongue, vallecula and anterior surface of the epiglottis, lateral wall including the tonsillar arches as far as possible). | Fails to visualize relevant structures. |  | Visualizes most relevant structures but in a hurried or “once over lightly” fashion. |  | Visualization of all relevant structures executed without negligence or omissions. |  |
| *(Optional)*  *Elicits specific movements when needed to visualize otherwise obscured areas (Base of tongue, vallecula or piriform recess).* | *Fails to elicit specific movements despite being relevant.* |  | *Requests movements but not enough to optimize visualisation.* |  | *Systematically elicits specific movements to optimize visualisation.* |  |
| To the extent possible, visualizes all mucosal surfaces of the hypopharynx (pharyngo-oesophageal junction (postcricoid area), piriform sinus and the posterior wall). | Fails to visualize relevant structures. |  | Visualizes most relevant structures but in a hurried or “once over lightly” fashion. |  | Visualization of all relevant structures executed without negligence or omissions. |  |
| To the extent possible, visualizes all mucosal surfaces of the larynx (supraglottic region including the inferior surface of epiglottis and aryepiglottic folds and close up visualization of the glottis). | Fails to visualize relevant structures. |  | Visualizes most relevant structures but in a hurried or “once over lightly” fashion. |  | Visualization of all relevant structures executed without negligence or omissions. |  |
| Elicits specific movements to assess vocal fold motion and functionality (adduction/abduction, closure, phonation, hyperventilation). | Fails to elicit movements to assess vocal fold motion and functionality. |  | Movements requested inadequate for full assessment of vocal fold motion and functionality. |  | Systematically elicits specific movements to fully assess vocal fold motion and functionality. |  |
| *(Optional)*  *When obvious pathology is found, deliberately attempts to identify borders of lesion.* | *Fails to examine pathology for delineation.* |  | *Visualizes parts of lesion for delineation but in a hurried or “once over lightly” fashion.* |  | *Systematically examine pathology for delineation.* |  |

**Section G: Governance**

1. Each pulmonology service performing FNL should have a written local protocol covering indications, consent, procedure, and complication management.
2. FNL activity and outcomes should be included in departmental quality‑improvement and audit programs.
3. Clear referral and communication pathways with ENT services should be in place for patients with abnormal or complex findings.
4. Access to FNL should be considered a core component of advanced pulmonology training in centres where services and supervision exist.
5. Barriers to FNL implementation should be routinely reviewed at departmental level.
6. Please describe key barriers or enablers for implementing high‑quality FNL services in pulmonology in your context.

**Section H: Future priorities**

1. Please rate the importance of future research on each of the following:
   a. Learning curves and minimum experience thresholds for FNL in pulmonologists.
   b. Validation of FNL procedure‑specific assessment tools.
   c. Patient‑reported outcomes and satisfaction with FNL performed in pulmonology clinics.
   d. Complication rates and safety of FNL in different clinical settings.
   e. Cost‑effectiveness and service models for integrating FNL into pulmonology pathways.
2. Please list any other priority research or implementation topics related to FNL in pulmonology.
